# Mitochondrial Gene Sequence (*COI*) Reveals the Genetic Structure and Demographic History of *Lymantria dispar* (Lepidoptera: Erebidae: Lymantriinae) in and around China

**DOI:** 10.3390/insects10050146

**Published:** 2019-05-22

**Authors:** Yao Xu, Sufang Zhang, Hongbin Wang, Mei Wang, Guohong Li

**Affiliations:** Key Laboratory of Forest Protection of State Forest Administration, Research Institute of Forest Ecology, Environment and Protection, Chinese Academy of Forestry, Beijing 100091, China; yaoxu208@163.com (Y.X.); zhangsf@caf.ac.cn (S.Z.); wangmeicnu@163.com (M.W.); liguohong@caf.ac.cn (G.L.)

**Keywords:** *Lymantria dispar* specimens, species-specific primers, *COI* gene, genetic structure

## Abstract

The gypsy moth, *Lymantria dispar*, is among the most destructive quarantine pests of forests. Here, we reconstructed the genetic structure and determined the population differentiation of gypsy moths across its distribution range at different times. This information could be used to both improve the prevention and detection of gypsy moths in the field. Using 31 newly designed species-specific primers targeting fragments of 216–1102 bp, we identified 103 full-length cytochrome oxidase subunit I (*COI*) gene sequences from eight fresh samples and 95 *L. dispar* specimens collected between 1955 and 1996, mainly in China. Combining 103 full-length *COI* gene sequences with 146 *COI* gene sequences from Genbank or DNA barcode libraries, we analyzed the genetic differentiation, gene flow and haplotypes between gypsy moth populations in order to reflect the genetic structure and population dynamics of gypsy moths. We discovered 25 previously unknown haplotypes from old gypsy moth specimens. We found that the genetic diversity among gypsy moth populations (collected in the same region at different time points) was relatively high. Furthermore, the genetic structure of Chinese geographical populations (Heilongjiang, Liaoning, Beijing) in different years was distinct. Our results suggested that some gypsy moths in China showed the genetic affinity with European gypsy moths (a sub-species of gypsy moths found mainly in Europe).

## 1. Introduction

The gypsy moth, *Lymantria dispar* (Linnaeus, 1758) (Insecta: Lepidoptera: Erebidae: *Lymantriinae*) [1,2,3], is among the most destructive pests of forests around the world (including Asia, Europe and North America), causing injury to approximately 400 deciduous and coniferous tree species [4,5,6,7]. Three sub-species have been identified based upon their morphological criteria, female flight capability and geographic origins: *Lymantria dispar dispar* Linnaeus, *Lymantria dispar asiatica* Vnukovskij and *Lymantria dispar japonica* Motschulsky [2]. *L. d. dispar* is found mainly in Europe, including most countries and regions in Western Europe, North Africa, France and North America. *L. d. asiatica* occurs from Central Asia to East Asia, including Russia, China and the Korean peninsula, while *L. d. japonica* is restricted to Japan (Honshu, Shikoku, Kyushu and Hokkaido) [8,9,10,11,12]. Subspecies *L. d. asiatica* and *L. d. japonica* have long been thought to pose greater global threats because of the superior flight ability of the females [13,14,15,16,17]. In addition, these adult females are also highly responsive to light and lay their eggs on the hulls of docked ships [18,19].

Over recent years, increased levels of international trade and tourism have facilitated the inadvertent dispersal of gypsy moths beyond their native ranges. However, introducing *L. dispar* to areas where it is not already present causes unpredictable effects upon the economic benefits and ecological stability of forest ecosystems [20]. For quarantine purposes, more and more studies are trying to investigate the genetic structure and demographic history of *L. dispar* over a wide geographic distribution by using various methods, such as allozyme detection, amplified fragment length polymorphism, restriction fragment length polymorphism, sequence-based analysis and microsatellites [21,22,23,24,25,26,27,28]. 

In the first such attempt, by Bogdanowicz et al. [22], three mitochondrial genes (*COI*, *COII*, *NDI*) (cytochrome oxidase subunit I, cytochrome oxidase subunit II, NADH dehydrogenase subunit I) were investigated in order to perform cluster analysis for gypsy moths from Japan, Europe, Tunisia and North America. This strategy revealed four populations: Okinawa in Japan; Hokkaido in Japan; Honshu and Kyushu in Japan and the Asian continent; Europe, Tunisia and North America. Later, Keena et al. [14] separated 46 gypsy moth strains into four groups (North American, European, Siberian and Asian) based on two restriction sites for cytochrome oxidase subunit I (*COI*), the nuclear FS1 marker and four microsatellite loci. 

More recently, Wu et al. [29] identified four genetic clusters within *L. dispar* by analyzing microsatellite loci and the mitochondrial DNA sequences of 1738 gypsy moths, with three clusters corresponding to the three known subspecies (*L. d. asiatica*, *L. d. dispar*, *L. d. japonica*); the North American populations formed a distinct fourth cluster. Based on the DNA barcode sequences of gypsy moth specimens sourced from 16 sites in China, Japan, Russia, Europe and the USA, Chen et al. [24] divided 288 specimens into two groups: an Asian group (individuals from China, the Far East of Russia and Japan) and a European group (individuals from Siberian Russia, Greece, Lithuania and the USA). Furthermore, using microsatellite loci and mitochondrial genes, the population genetic structure and demographic history of 20 *L. dispar* populations from Asia were analyzed by Kang et al. [30], and revealed three groups: Group 1 (Korean inland region and adjacent areas); Group 2 (Korean southern region) and Group 3 (Hokkaido region). Research on population genetic structure provides insight for enhancing monitoring and exclusion programmes [24,29].

In most studies, however, only recently formed population genetic structures have been revealed; consequently, we know little about the precise history of gypsy moth populations in the field. In light of this, further investigations are required to identify the historical genetic structure and demographic history of *L. dispar*, a subspecies that is particularly prone to taxonomic confusion. For example, some individuals of *L. dispar asiatica* that were collected from the southern coastal area of Korea reportedly had morphological characteristics similar to those of *L. d. japonica* [31]. Chen et al. [24] further suggested that gypsy moths from Guizhou, plus one from Liaoning, exhibited genetic affinity with the Siberian population, and might even be a new cryptic subspecies of *L. dispar* [24]. 

To resolve this taxonomic confusion between gypsy moth subspecies, a demographic history of *L. dispar* populations based on intensive sampling was deemed necessary. To this end, we investigated the genetic structure of gypsy moth specimens (collected over the past 10 years or even decades ago) in several regions of the world, by using their *COI* (cytochrome oxidase subunit I) sequences. We also used several dried and formalin-fixed specimens, collected between 1955 and 1996, instead of fresh samples. Worldwide, museums and other natural history collections, house millions of animal and plant specimens, making these institutions a treasure trove of biological diversity [32]. The combination of fresh and museum specimens has created an incredible potential for advanced studies of evolutionary change [33,34,35]. For example, utilizing thess types of material, researchers recently revealed some remarkable instances of phenotypic or genotypic change over short timescales (years) in response to strong selective pressures [36,37]. Among the many opportunities provided by combining genomic and morphological data from the same specimens, most earlier studies have focused upon the DNA fragment size of biological collections, species delimitations, thus facilitating the deduction of phylogenetic associations and the construction of DNA barcode sequence libraries for DNA-based identifications [38,39,40,41,42,43,44,45,46,47,48,49,50]. 

Building upon this foundation, in the present study, we have attempted to link molecular data from museum specimens, as well as recently collected fresh samples in order to assess the genetic associations of these specimens. In short, this study made use of a large number of specimens which had been collected over the past 53 years (1955–2008). Importantly, these included many specimens taken at the earliest known time of gypsy moth distribution in China, as well as specimens collected from the USA in 1996. All *COI* gene sequences of gypsy moth specimens were amplified and spliced; along with the *COI* gene sequences of gypsy moths deposited in GenBank or DNA barcode libraries, we hoped to enhance our understanding of gypsy moth dynamics. For instance, our aim was to use this robust dataset to characterize the variation in the genetic structure of populations over different years and genetic differentiation in populations across different years. We particularly focused upon the mitochondrial cytochrome oxidase subunit I (*COI*) gene, which has been successfully used in the past to investigate phylogenetic relationships in the three *L. dispar* sub-species and widely used to study the genetic structure and genetic differentiation of the gypsy moth populations [24,29,30].

## 2. Materials and Methods

### 2.1. Samples

In 2017, we obtained fresh larval samples from the Insect Virus Research Centre of the Chinese Academy of Forestry. Several gypsy moth egg masses were collected in Liaoning Province in 2008 (Table 1), and when hatched, reared on an artificial diet in a greenhouse under controlled conditions (temperature: 24–26 °C; photoperiod: 14-h-light:10-h-dark and a relative humidity of 60%–80%). Samples were stored in 100% ethanol and kept at –20 °C to await extraction.

**Figure 1:**
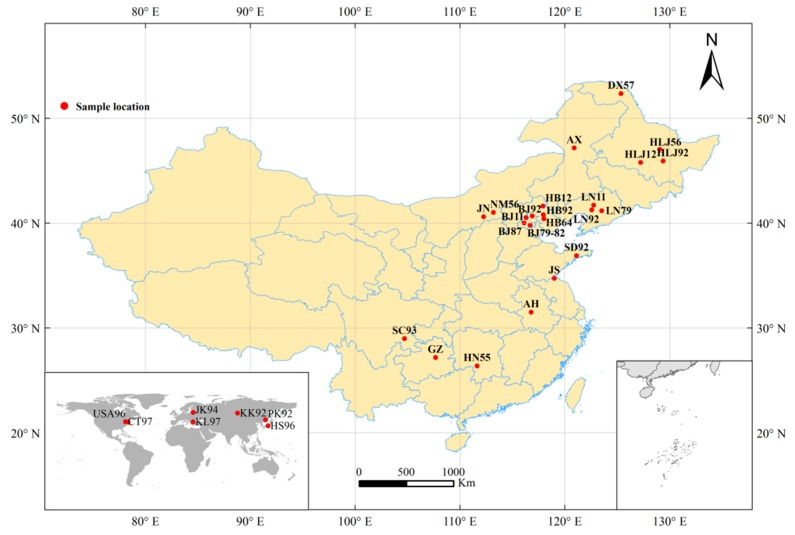
The collection sites of *L. dispar.* Populations labels follow those in Table 1 and Table S2. The collection locations of HLJ56 and HLJ79 populations are the same. The collection locations of LN79 and LN08 populations are the same. BJ79, BJ81 and BJ82 populations are collected in the same area.

A total of 75 dried gypsy moth specimens, and 20 formalin-fixed larval specimens, were analyzed (Table 1). All of the specimens collected between 1955 and 1996 from 10 different locations had been preserved at ambient temperature, without dehumidification, in the Specimen Banks of Forest Insects at the Research Institute of Forest Ecology, Environment and Protection (Chinese Academy of Forestry, China). Adult specimens were dried naturally before setting them with an insect pin, whereas larvae were preserved in formalin. Most of the formalin-fixed specimens were kept together in a single large jar. A few specimens had been stored in cotton-stoppered glass vials, with several vials kept together submerged in formalin within a larger jar. Figure 1 shows a map of sample collection points.

### 2.2. DNA Extraction

All laboratory equipment and work areas were first cleaned with 75% ethanol to limit potential contamination before extraction. We also used new consumables and reagents to prevent possible cross-contamination of DNA extraction from other species. All specimens were briefly treated to remove surface contamination before DNA extraction. For fresh samples, larval samples were first washed with sterile distilled water, and part of their abdomen was ground in liquid nitrogen. Sterilized brushes were used to remove bristles and dust from the old dried specimens prior to DNA extraction. All dried specimens were also washed in sterile distilled water to remove surface contamination, and then air-dried. For dried specimens, part of their abdomen was ground in liquid nitrogen, without causing any damage to other external morphological features. The ground tissue (40 mg) of both fresh samples and dried specimens was then used for DNA extractions. The tissue was then lysed, and the lysate purified with an E.Z.N.A.™ Insect DNA kit (Solarbio, Shanghai, China) in accordance with the manufacturer’s instructions, but with a slight modification, i.e., lysate was incubated at 60 °C for 2 h.

For formalin-fixed larval specimens, another DNA isolation protocol was required. Approximately 60 mg of tissue from each specimen was cut into pieces and centrifuged at 10,000 g for 2 min. The supernatant was then discarded. Tissues were then rinsed overnight in xylene at room temperature (10–25 °C), centrifuged at 10,000 g for 1 min and the supernatant was discarded. Next, all tissue samples were treated with a gradient of ethanol concentrations (65%, 70%, 75%, 80%, 85%, 90%, 95% and 100%) for 2 hours, and centrifuged at 5000 g for 10 min to gradually break protein-DNA cross-linkages caused by the long-term exposure to formalin. Lastly, the specimens were left to dry overnight at room temperature (10–25 °C). After completing this drying process, the E.Z.N.A.™ Insect DNA kit was used in accordance with the manufacturer’s instructions, but with a slight modification: lysate was incubated at 60 °C for 2 h. Extracted DNA was stored in an elution buffer (EB) at –20 °C. Samples from different years were individually extracted, to eliminate any cross-contamination between DNA extracts. After extractions, a DS-11 Spectrophotometer (DeNovix) was used to determine the DNA concentration and absorbance ratio (A260/A280) of each sample. Generally, the expected ratios for extracted DNA samples from insects should range from 1.7 to 2.0 according to the manufacturer’s protocol for the E.Z.N.A.™ Insect DNA kit.

### 2.3. Primer Design, PCR Amplification and Sequencing

The sizes of extracted DNA fragments were determined by automatic capillary electrophoresis, with an observed fragment distribution of 21–900 bp across all specimens. Based upon these results, and full *COI* sequences from *L. dispar* (FJ617240, KY798442, KY923059–KY923067), 31 new species-specific primers (Table S1) were designed using the Primer v5.0 tool. The size of target fragments ranged from 216 to 1102 bp. Full *COI* gene sequences were recovered via the combination of various overlapping amplicons.

All PCR amplifications were performed directly on total DNA extracts, in a final volume of 25 µL, consisting of 12.5 µL of 2× Tap PCR MasterMix (0.1 U Taq Polymerase/µL, 500 µM dNTP each, 20 mM Tris-HCl (pH 8.3), 100 mM KCl, 3 mM MgCl_2_, with other stabilizers and enhancers), 1 µL of each primer (10 µM), 1 µL of DNA Template (25.0–125.0 ng/µL), with ddH_2_O added to 25 µL. The PCR conditions were as follows: 3 min at 94 °C; followed by 30 cycles for 30 s at 94 °C, 30 s at 48.0–56.8 °C (the annealing temperatures were different for certain primer pairs, see Table S1), 1 min at 72 °C; and with a final extension step of 5 min at 72 °C. Amplifications were performed using a Gene-Explorer Touch Gene Amplifier (GE9612T-S, Hangzhou Bio-Gener Technology Co, Ltd). Thereafter, 3 µL of each PCR product was resolved by 1.0% agarose gel electrophoresis to determine whether the amplification had succeeded; and the remaining reaction volume was used for Sanger sequencing. The DNA sequencing was performed using bidirectional reads in a semi-automated sequencing process based on magnetic beads (TSINGKE Biological Technology, Beijing, China).

All sequences for a given specimen were first aligned separately in BioEdit v7.2 software [51] and then connected to generate its *COI* sequence. To verify the authenticity of *COI* sequences obtained from old specimens, we crosschecked the new sequences with established reference sequences (FJ617240, KY798442, KY923059–KY923067).

### 2.4. Gypsy Moth Mitochondrial COI Sequence Data

The *COI* gene sequences of gypsy moths, from various countries and different sites, were previously amplified and uploaded into Genbank or DNA barcode libraries (Bold Systems V4). Such sequences can provide crucial information for defining genetic structure and demographic history in terms of its area of origin and adjacent areas [24,29,30]. In the present study, we obtained the *COI* gene sequences of gypsy moth specimens collected during different periods. Combining this information with the *COI* gene sequences of gypsy moths published in Genbank or the DNA barcode library (Bold Systems V4), we identified the genetic structure of gypsy moths in specific periods and discussed associated changes in genetic structure at different times. Therefore, in order to reveal the historical genetic structure of gypsy moths, in addition to the specific gene sequences obtained in this study, we also downloaded the following gene sequences from Genbank or the DNA barcode library (Bold Systems V4): (1) 11 full-length *COI* gene sequences from gypsy moths from around the world (FJ617240, KY798442, KY923059~KY923067), combined with the *COI* gene sequences of gypsy moths obtained in this study, both of which may perhaps reveal previously undetected haplotypes; (2) 86 *COI* gene sequences from gypsy moths in China (GMCF001-14~GMCF074-14, HM775684~HM775692, HM775605, HM775607, HM775608) combined with the *COI* gene sequences of gypsy moths obtained in the present study, which could similarly reveal previously undetected haplotypes of the Chinese gypsy moth populations; (3) 63 *COI* gene sequences collected from Japan, Russia, the USA, Greece, Lithuania and different regions of China in the 1990s (GMCF075-14, GMCF085-14~GMCF134-14, HM775684~HM775692, HM775605, HM775607, HM775608), which revealed the genetic structure and population differentiation of the gypsy moth population in the 1990s; and (4), recently published *COI* gene sequence from Chinese gypsy moth populations (GMCF001-14~GMCF074-14). It was hoped that these sequences (including *COI* gene sequences of the gypsy moth specimens) could reveal temporal changes in the genetic structure of the gypsy moth population. The information of all the downloaded gene sequences is shown in Table S2, and the collection locations for all sequences are marked in Figure 1.

Compared with the recently published datasets from fresh samples, the gene sequences used in this study provide additional valuable information relating to species history. However, this heterochroneous dataset, which features population-level information spanning 53 years (1955–2008), intrinsically violates the assumption of constant sampling time; hence, heterochrony had to be explicitly avoided for in further analyses. To remove such heterochrony-driven bias, the *COI* gene sequences obtained during the same time period were correspondingly assigned to the same group according to the time of collection. For instance, 28 *COI* gene sequences from the HN55, HLJ56, NM56 and DX57 populations were used to analyze the genetic structure among four geographic populations in the 1950s. Similarly, 18 *COI* gene sequences from three regions (Heilongjiang, Liaoning and Beijing) were used to assess genetic structure among these three populations in the 1970s. Combining this data with *COI* gene sequences from the 1990s and 2010s from Heilongjiang, Liaoning and Beijing, enabled us to analyze changes in genetic structure across different years. Furthermore, we had hoped that DNA barcode sequences from the same region, but from different years, may well provide information relating to the population dynamics of gypsy moths in that region. For example, using this model, we analyzed the *COI* sequences for the Beijing populations over several years (1979, 1981, 1982, 1987, 1992, 2011).

### 2.5. Analysis of Genetic Structure in Populations of Gypsy Moths

Genetic differentiation coefficients (*F*_ST_) and gene flow (Nm) are two key indices that reflect genetic structure. The gene differentiation coefficient is often used to reveal variation in allele frequency among different populations, along with the degree of genetic differences between disparate species or populations, which is an important parameter that expresses the evolutionary rate of colonies. As gene flow can maintain genetic similarity between populations through the migration of gametes, individuals, or even entire populations, this represents a key factor in the homogenization of population genetic structure. Generally, the species populations with high gene flow have a lower degree of genetic differentiation, whereas populations with low gene flow will exhibit greater genetic differentiation. According to gene flow studies, genetic drift becomes the main factor driving genetic differentiation among populations when Nm < 1. In contrast, when Nm > 4, gene exchange between populations is sufficient to counteract the effects of genetic drift, thereby preventing genetic differentiation between populations [52]. Hence, pairwise comparisons of *F*_ST_ values and Nm values between *L. dispar* populations were calculated using the Kimura two-parameter model in Arlequin v3.0 (alpha level of significance = 0.05, significance level obtained from *n* = 1000 permutations) [53] based on its *COI* gene sequences.

The median-joining haplotype network can be useful for bothconveying population structures and estimating the genealogical relationships among various haplotypes. For this reason, a median-joining network was also derived based on the *COI* gene sequences of gypsy moths, using the program NETWORK v4.6.1.3 [54]. Moreover, for a specific species, its evolutionary potential and adaptability to the environment are closely related to genetic diversity of the populations. Those species endowed with higher genetic diversity are more able to tolerate and survive environmental stresses; hence, values for the number of nucleotide differences (K), nucleotide divergence (Pi) and haplotype diversity (Hd) were computed with DnaSP software [55].

### 2.6. Phylogenetic Analyses among Populations of Gypsy Moths

Phylogenetic trees were constructed using the neighbor-joining (*NJ*) phylogenetic approach in MEGA v7.0 software [56], using the *COI* sequence from *Lymantria albescens* (Genbank ID: NC035627) serving as an outer group. The best evolutionary model was chosen, and its phylogeny was tested by the bootstrap method. Rates of change among lineages were presumed to be gamma distributed (G), and the bootstrap of each branch of the phylogenetic tree was subjected to *n* = 1000 replications.

## 3. Results

### 3.1. Assessing the Utility of Newly-Designed Primer Pairs

For dried specimens, a total of 3–12 µg DNA was normally extracted from a 40 mg tissue sample, with an absorbance ratio (260/280) of 1.8–2.0. DNA extracts from formalin-fixed specimens exhibited a DNA concentration of 18.7–28.3 ng/µL; here 260/280 ratios mostly ranged from 1.8 to 2.0. These results indicated that DNA extracts for the majority of samples were pure and could be sequenced. To do this, 31 newly designed primer pairs were tested with fresh samples obtained in 2018; 30 of these successfully generated *COI* sequences and only Lco31, targeting fragments of 1102 bp, failed to yield a DNA sequence. This showed that the designed primers were reasonably reliable for further experimentation. Not all primers amplified the target band in all specimens, but for all specimens, we were able to assemble the complete *COI* gene sequence from the amplified gene sequences. In all, a total of 103 full-length *COI* gene sequences were recovered by assembling fragments with overlapping sequences. All DNA sequences are listed in Suplementary Materials.

### 3.2. New Haplotypes from Old Gypsy Moth Specimens

**Figure 2:**
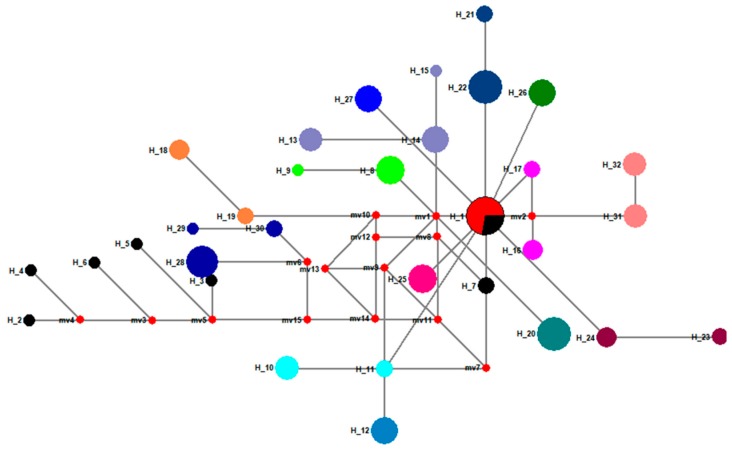
Median-joining haplotype network for full-length cytochrome oxidase subunit I (*COI*) gene sequences of *L. dispar.* Haplotypes 1 to 7 had already been published. The haplotype of a population is represented by the same color. The size of the fan area corresponds to the proportion of each population with the same haplotype. Each link between haplotypes represents one mutational difference. MV nodes indicate that inferred haplotypes were not found in the sampled populations. The detailed information of the haplotypes distribution is shown in Table 2.

**Figure 3:**
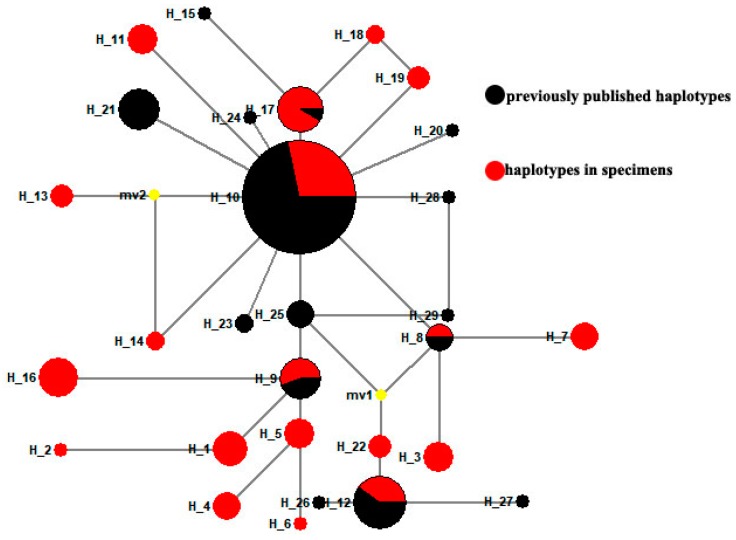
Median-joining haplotype network for DNA barcode sequences of *L. dispar.* Previously published haplotypes are shown in black type, while those haplotypes obtained from the old museum specimens are in red. Each link between haplotypes represents one mutational difference. MV nodes indicate that inferred haplotypes were not found in the sampled populations. The detailed information of the haplotypes distribution is shown in Table 3.

In a parsimonious network built with 103 full-length *COI* gene sequences obtained from old specimens and 11 published *COI* gene sequences, we discovered 25 new haplotypes (Figure 2). Comparing these haplotypes among regional populations in the 1950s, 1970s or 1990s, revealed that they were geographically distinct (Figure 2). Moreover, regional populations from the same location, but collected at different times, also showed clearly distinctive haplotypes (Figure 2). Fourteen previously unknown haplotypes for Chinese gypsy moth populations were revealed (Figure 3), based on the 95 DNA barcode sequences obtained in this study and those 86 that had already been published. In the median-joining network, one high-frequency haplotype (H10, 67 samples) was connected by low-frequency haplotypes (Figure 3). We also identified five shared haplotypes between the *COI* gene sequences obtained in our study and previously published data. Notably, a shared haplotype existed between the SC93 (Sichuan in 1993), LN11 (Liaoning in 2011) and GZ (Guizhou in 2011) populations. Chen et al. [24] indicated that the mitochondrial *COI* genotype of LN11 and GZ populations was similar to that of the Siberian gypsy moth.

### 3.3. Genetic Differentiation in Gypsy Moth Populations across Different Years

**Figure 4:**
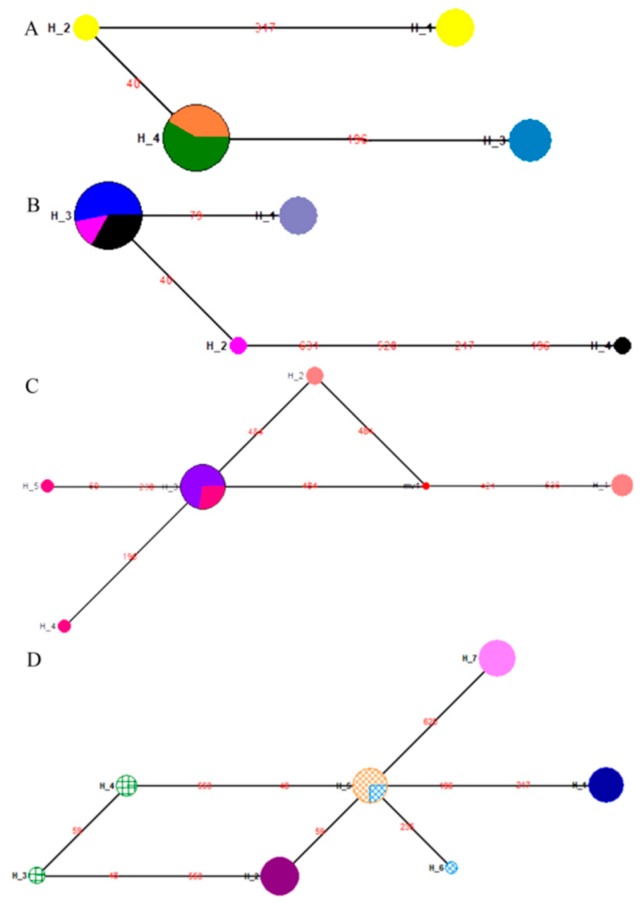
Median-joining haplotype network of *L. dispar* based on the mtDNA *COI* gene. (**A**) All haplotypes for Heilongjiang populations. H1: HLJ56(4); H2: HLJ56(2); H3: HLJ79(5); H4: HLJ92(5), HLJ12(7). (**B**) All haplotypes for Liaoning populations. H1: LN79(5); H2: LN92(1); H3: LN92(2), LN08(8), LN11(5); H4: LN11(1). (**C**) All haplotypes for Hebei populations. H1: HB64(3); H2: HB64(2); H3: HB92(3), HB12(8); H4: HB12(1); H5: HB12(1). (**D**) All haplotypes for Beijing populations. H1: BJ79(8); H2: BJ81(10); H3: BJ82(2); H4: BJ82(3); H5: BJ87(8), BJ92(2); H6: BJ92(1); H7: BJ11(9). The size of the fan area corresponds to the proportion of each population with the same haplotype. The number of samples included in each haplotype is given in brackets. Each link between haplotypes represents one mutational difference. MV nodes indicate that inferred haplotypes were not found in the sampled populations. The detailed information of population codes is shown in Table S2.

Specimens from the four years 1956, 1979, 1992 and 2012 were obtained to evaluate the variation in *COI* sequence over time and to provide insight into gypsy moth dynamics in Heilongjiang, China. We first analyzed the genetic diversity among Heilongjiang populations, and found that the mean number of nucleotide differences (K), haplotype diversity (Hd) and nucleotide diversity (Pi) were 1.0593, 0.6719 and 0.0016, respectively. In addition, four haplotypes were identified in the Heilongjiang population (Figure 4A). A shared haplotype was revealed between the HLJ92 and HLJ12 populations; however, none could be found in the HLJ56, HLJ79, HLJ92 and HLJ12 populations (Figure 4A). Genetic differentiation between the HLJ92 (collected in 1992) and HLJ12 populations (collected in 2012) was similar (*p* > 0.05), whereas it differed significantly among the other populations (*p* < 0.01, Table S3).

Analysis of genetic diversity among the Liaoning populations from the four years 1979, 1992, 2008 and 2011 revealed that mean K, Hd and Pi values were 0.9048, 0.5022 and 0.0014, respectively. Additionally, four haplotypes were identified for the Liaoning populations (Figure 4B) but no shared haplotype was identified between the LN79 population and other populations. Lastly, genetic differentiation between LN11 population and LN92, LN08 populations was not significant (Table S4). For three Hebei populations (1964, 1992 and 2012), mean K, Hd and Pi values were 1.3856, 0.6744 and 0.0021, respectively, with five haplotypes discovered (Figure 4C). Two haplotypes found in the HB64 population (collected in 1964) were previously unknown. With regards to genetic differentiation, *F*_ST_ values between the HB92 population and the HB64, HB12 populations were similar (*p* > 0.05, Table S5), while those of the HB64 population differed significantly from the HB12 population (*p* < 0.05, Table S5).

DNA barcode sequences of Beijing populations corresponding to the six years 1979, 1981, 1982, 1987, 1992 and 2011 were analyzed; mean K, Hd and Pi values were 1.9073, 0.8281 and 0.0029, respectively. Overall, there was relatively high nucleotide polymorphism and genetic diversity in the Beijing populations over different years, for which seven haplotypes were identified (Figure 4D). The four haplotypes found in old specimens were previously unknown. Notably, there was a shared haplotype between the BJ87 (collected in 1987) and BJ92 populations (collected in 1992), and both populations showed similar levels of genetic differentiation (*F*_ST,_ *p* > 0.05); however, the other populations (except for the BJ87 and BJ92 populations) showed significantly genetic differentiation (*p* < 0.01.; Table S6). Moreover, the genetic differentiation (*F*_ST(average)_ = 0.9293) between the BJ79 population (collected in 1979) and other populations was the greatest, but the lowest (*F*_ST (average)_ = 0.6078) was observed between the BJ87 population and other populations (Table S6).

### 3.4. Variation in Population Structure for Three Geographical Populations over Different Years

For the gypsy moth populations in Liaoning and Beijing, China, we obtained DNA barcode sequences from moth samples collected in 1979, 1992 and 2011. In addition, we obtained DNA barcode sequences of the Heilongjiang population sampled in 1979, 1992 and 2012. Using these gene sequences, we analyzed the differences in genetic diversity in three gypsy moth populations over three years (1979, 1992, 2011 or 2012). Although the collection times of the three populations were not consistent, the differences in gene sequence were almost negligible because of the small difference between the two collection times (2011 and 2012). For Liaoning populations, mean K, Hd and Pi values were 1.3297, 0.659 and 0.0021, respectively. For Beijing populations, mean K, Hd and Pi values were 1.6316, 0.658 and 0.0025, respectively. For Heilongjiang populations, mean K, Hd and Pi values were 0.4412, 0.441 and 0.0007, respectively.

**Figure 5:**
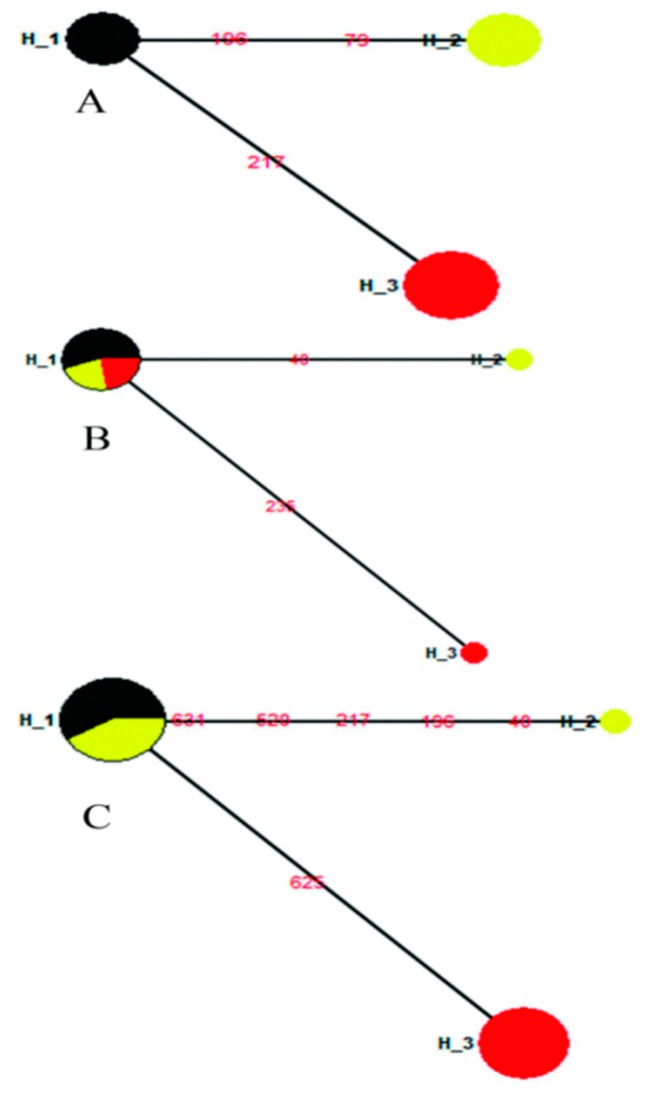
Median-joining haplotype network of three populations across different years. (**A**) All haplotypes for the three geographical populations in 1979. H1: HLJ79(5); H2: LN79(5); H3: BJ79(8). (**B**) All haplotypes for the three geographical populations in 1992. H1: HLJ92(5), LN92(2), BJ92(2); H2: LN92(1); H3: BJ92(1). (**C**) All haplotypes for the three geographical populations in 2011. H1: HLJ12(7), LN11(5); H2: LN11(1); H3: BJ11(9). The size of the fan area corresponds to the proportion of each population with the same haplotype. The number of samples included in each haplotype is given in brackets. Each link between haplotypes represents one mutational difference. The detailed information of population codes is shown in Table S2.

In addition, we analyzed the changes in genetic structure of three geographical populations across the three periods. For these three geographical populations sampled in 1979, mean *F*_ST_, K, Hd and Pi were 0.9279, 1.3726, 0.6863 and 0.0021, respectively. The differences in *F*_ST_ values among the three populations were significant (*p* < 0.05, Table S7), with no evidence of a shared haplotype in 1979 (Figure 5A). In 1992, mean *F*_ST_, K, HD and Pi were 0.4309, 0.3636, 0.3455 and 0.0006. *F*_ST_ values between the BJ92 population and both the HLJ92 and LN92 populations were not significant (*p* > 0.05, Table S8) and shared one haplotype (Figure 5B). For 2011, the three populations exhibited means of 0.5496, 0.9610, 0.5584 and 0.0015, respectively; the HLJ12 and LN11 populations showed not significantly difference in terms of *F*_ST_ (*p* > 0.05, Table S9), with one shared haplotype between two populations (Figure 5C).

### 3.5. Population Structure of the Four Geographical Populations in the 1950s

**Figure 6:**
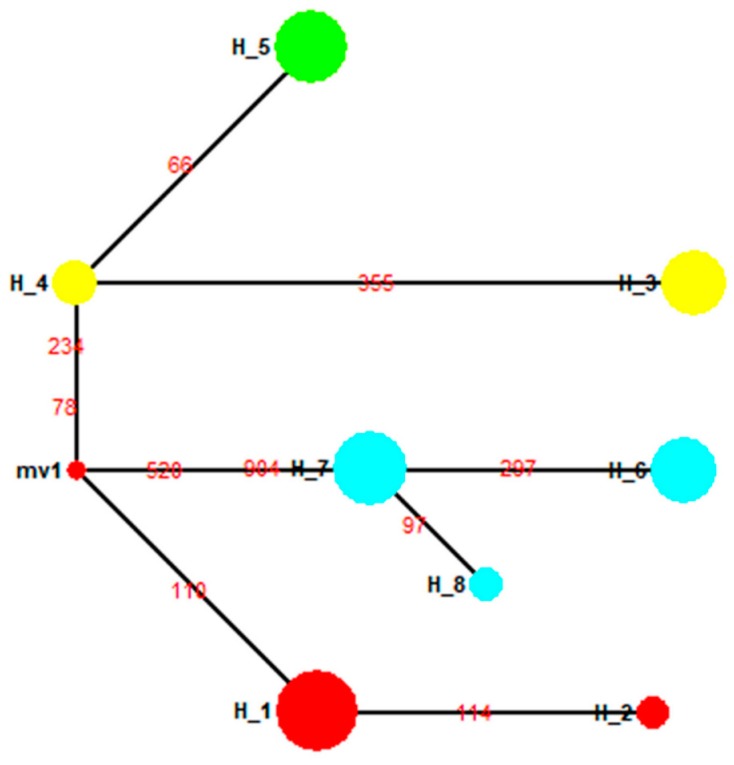
Median-joining haplotype network of *L. dispar* populations in the 1950s. All haplotypes of the four geographical populations (Hunan, Heilongjiang, Inner Mongolia and Greater Hinggan) in the 1950s. H1: HN55(6); H2: HN55(1); H3: HLJ56(4); H4: HLJ56(2); H5: NM56(5); H6: DX57(4); H7: DX57(5); H8: DX57(1). The haplotype of a population is represented by the same color. The size of the fan area corresponds to the proportion of each population with the same haplotype. The number of samples included in each haplotype is given in brackets. Each link between haplotypes represents one mutational difference. MV nodes indicate that inferred haplotypes were not found in the sampled populations. The detailed information of population codes is shown in Table S2.

We investigated population structure in Hunan, Heilongjiang, Inner Mongolia and Greater Hinggan, based upon *F*_ST_ and Nm values, which are shown in Table S10. In the 1950s, these four geographical populations showed relatively high genetic differentiation, with values ranging from 0.8847 to 0.9356, and highly significant differences (*p* < 0.01) (Table S10). The Nm values among the four populations were <1 (0.0344–0.0652) (Table S10), which was consistent with their high levels of differentiation. In addition, eight haplotypes were identified for these populations in the 1950s (Figure 6).

### 3.6. Population Structure for Geographical Populations in the 1990s

**Figure 7:**
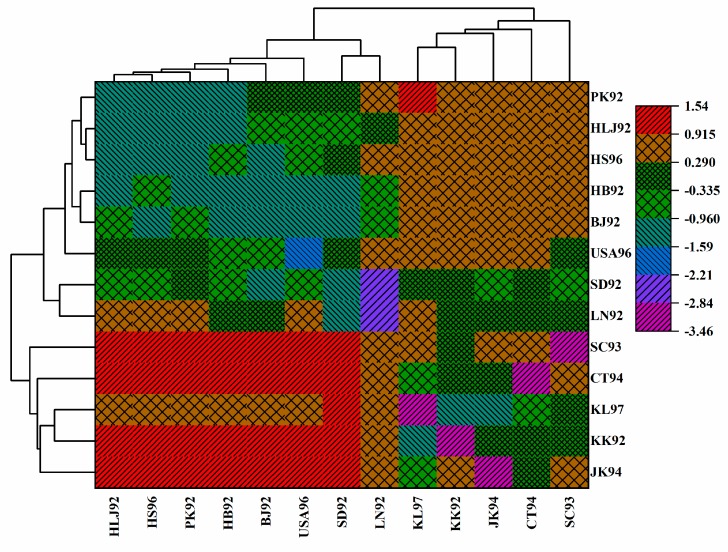
A cluster heat map reflecting the genetic differentiation between populations.

**Figure 8:**
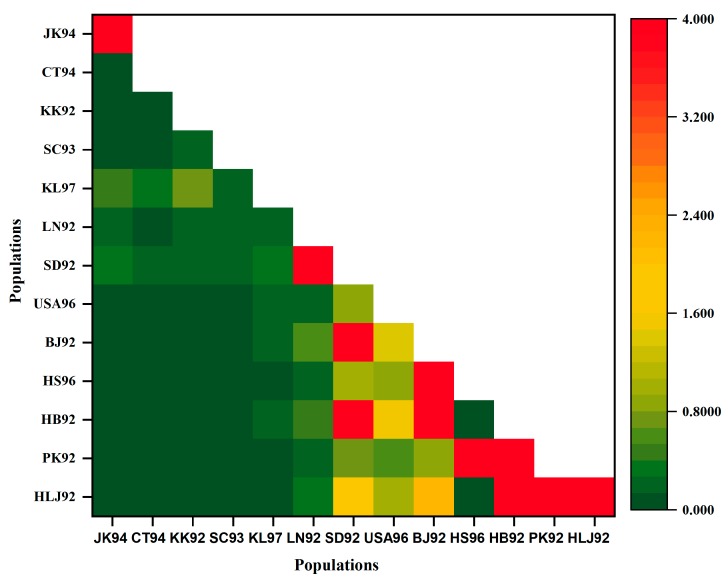
A heat map reflecting the gene flow between populations. It is not possible to clearly show the gene flow between all populations in the heatmap due to the large difference in Nm values between populations (0–63). Moreover, it indicates that the degree of gene flow between populations is strong when Nm > 4. Therefore, we set gene flow values (Nm) to 4 when Nm > 4 in the heatmap.

A total of 85 DNA barcode sequences, including 62 *COI* gene sequences downloaded from GenBank and Bold Systems V4, from 13 regions in the 1990s, were obtained in this study. With these, the genetic structure and demographic history of gypsy moths in China, and adjacent areas, were revealed. Firstly, we calculated pairwise genetic differentiation coefficients (*F*_ST_) and gene flow (Nm) between all populations; values for the 1990s are shown in Table S11. Two cluster heat maps were also generated to reflect the degree of genetic differentiation and gene flow between populations (Figure 7, Figure 8). In the cluster heat map, the SC93, CT94, KL97, KK92 and JK94 populations were clustered together and the degree of genetic differentiation among those populations was relatively low. Furthermore, the HLJ92, BJ92, USA96, HB92, SD92, LN92, HS96 and PK92 populations were clustered together and the degree of genetic differentiation between populations was also relatively low (Figure 7). In the heat map of gene flow, we showed that the gene flow was strong between the SD92, USA96, BJ92 populations and the HLJ92, PK92, HB92 and HS96 populations (Figure 8).

In a cluster heat map, all *F*_ST_ values are standardized according to the following formula, i.e.,

(1) z=(x−u)σ,

x: *F*_ST_ values between populations, u: Mean of all *F*_ST_ values, σ: Standard deviation of all *F*_ST_ values. The degree of genetic differentiation between populations is positively correlated with the z-value.

**Figure 9:**
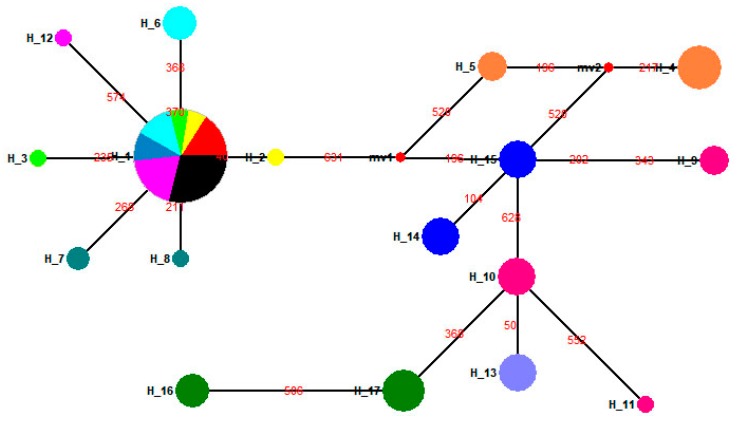
Median-joining haplotype network of *L. dispar* populations in the 1990s. The haplotype of a population is represented by the same color. The size of the fan area corresponds to the proportion of each population with the same haplotype. Each link between haplotypes represents one mutational difference. MV nodes indicate that inferred haplotypes were not found in the sampled populations. The detailed information of the haplotypes distribution is shown in Table 4.

**Figure 10:**
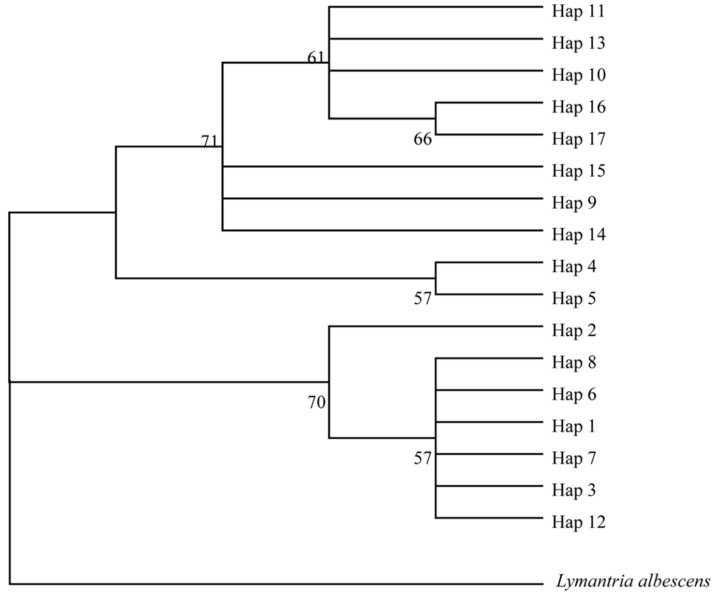
A phylogenetic tree for all gypsy moth haplotypes in the 1990s. The *Lymantria albescens COI* gene served as a basal outlier group. Numbers on the tree represent confidence values. The detailed information of the haplotypes distribution is shown in Table 4.

We identified 17 haplotypes for gypsy moths in the 1990s (Table 4). In this network, two major haplotypes (H1, H15) were connected to each other by the haplotype H2, indicating delineation between the two groups (Figure 9). Group 1 contained seven haplotypes and consisted of individuals from China (except the SC93 population), Honshu (Japan), Primorsky Krai (Russia), and Newark, Delaware (USA, Figure 9). Group 2 included samples from Sichuan (China), Krasnoyarsk Krai (Russia), Kavala (Greece), Juodkrante (Lithuania) and Connecticut (USA) (Figure 9). A neighbor-joining (NJ) phylogenetic tree, based on the T92 + G model, was constructed for all haplotypes (Figure 10). Evidently, all haplotypes from HLJ92, LN92, BJ92, SD92, HB92, HS96, PK92 and USA96 populations were clustered together, while all those from the SC93, KL97, JK94, KK92 and CT94 populations were clustered into a single group (Figure 10).

## 4. Discussion

In previous studies, a large proportion of research focusing on the analysis of haplotypes from several geographic populations of gypsy moth has relied on fresh samples. In the present study, we analyzed the haplotypes of several gypsy moth populations by using museum specimens collected in different eras. We first supplemented the *COI* gene sequences of the gypsy moth specimens from more than a decade, or even several decades. These data would be used to supplement existing data for future evolutionary research of gypsy moths. In addition, our findings provide information and insight into the historical haplotypes of different geographic populations of gypsy moth, which revealed many previously unknown gypsy moth haplotypes. The abundance of haplotypes may be related to the strong adaptability of gypsy moths to changing environments. We also found that haplotypes of the gypsy moth populations in different periods were different; this may be related to genetic flow between different populations or the genetic diversity of gypsy moths under environmental pressure. In short, the extensive variation in haplotype observed in this study may complicate efforts to control and detect gypsy moths.

Herein, we provide the first report on the gypsy moth dynamics in Heilongjiang, Liaoning, Hebei, and Beijing based on DNA barcode sequences from different years (1956–2012). We found that nucleotide polymorphism and genetic diversity among these four populations had, at different time points, remained relatively high, indicating that substantial genetic variability exists among these populations collected in the same region at different time points. Probably some of this variability relates to environmental changes that occurred between 1956 and 2012. For example, elevated atmospheric CO_2_ and O_3_ concentrations, rising temperatures, and an uneven distribution of rainfall has been found to profoundly impact upon the composition of insect communities and the interactions between host plants and pest insects, and their natural enemies [57]. To successfully adapt to different environmental conditions, it argues that the long-term accumulation of genetic variation has led to significant genetic differentiation among populations, such that more genetically diverse species are more likely to persist under environmental stresses.

Furthermore, our results clearly showed that genetic diversity in Beijing populations was higher than that in the Liaoning population and the Heilongjiang population. From this, we inferred that the Beijing populations may be more adaptable to different ecological environments and had a strong flight characteristic. Therefore, we should pay more attention to the prevention and control of the gypsy moth populations with high adaptability and strong flight ability. Such information could be useful in detecting and preventing of gypsy moth outbreaks, for which it may be necessary to tailor monitoring and control programs in different regions based on their historical information derived from these. Furthermore, the degree of genetic differentiation between populations seems to be inconsistent with the time gaps between the sample collections in our study. For instance, genetic differentiation (*F*_ST(average)_ = 0.9732) between the HLJ79 population and other Heilongjiang populations was greater than that between the HLJ56 population and other Heilongjiang populations (Table S3). We inferred from these findings that gypsy moth populations carrying similar genetic information to the HLJ56 population re-invaded the Heilongjiang region in 1992 or 2012; in other words, these populations experienced genetic exchanges with the Heilongjiang populations in 1992 or 2012; thus, compared with the HLJ79 population, the genetic differentiation between the HLJ56 population and other Heilongjiang populations was relatively low.

A number of studies have revealed the genetic structure of gypsy moths in some areas [24,30], and provided guidance for the quarantine of moths between different regions and countries. In our study, we found that the population structure in different years was distinct among all three geographical populations (Heilongjiang, Liaoning and the Beijing populations). Genetic differentiation was greatest in 1979, but much lower in 1992 and 2011 (2012). Correspondingly, the median-joining haplotype network in different years was also distinct. Since we obtained gypsy moth specimens collected before 1979, we supposed that moths invaded these three regions sometime before 1979. Then, following infestation, genetic variation increased such that the gypsy moth population could adapt to the local environment. The environment differs across these three regions; as such, the genetic variation of the gypsy moth population is also different, such that the genetic differentiation of the three populations was higher in 1979. Doubtless, ongoing transportation, trade, and the development of tourism after this date were conducive to the dispersion of moths among the three regions. Genetic differentiation of the three populations had gradually decreased by 1992 and 2011 (2012). In addition, population structures between different geographical locations, changed at different times, a finding perhaps related to the long-range flight ability of adult females.

The genetic structure of gypsy moth populations from China, Japan, Russia, USA, Greece and Lithuania in the 1990s was also revealed in this study. We showed that the genetic distances of Chinese gypsy moth populations (HLJ92, BJ92, HB92, SD92 and LN92) were relatively close, probably as a consequence of their close geographical distance. This indicated that the gypsy moth populations were likely to spread between adjacent areas, a finding which also directly relates to the strong flight ability of the Asian moths. In addition, based on the genetic differentiation and gene flow between populations, we suspected that increasing levels of international trade and tourism in the 1990s facilitated the overseas dispersal of gypsy moth populations from Honshu (Japan), Primorsky Krai (Russia) and China.

Chen et al. [24] indicated that the mitochondrial *COI* genotype of two Chinese populations (Guizhou and Liaoning) was similar to that of the Siberian gypsy moths, though not identical. Our study re-visits the issue of whether there is genetic affinity between some of the gypsy moths from China and those found in Siberia [24]. In a neighbor-joining (NJ) phylogenetic tree constructed for all haplotypes in the 1990s, we found that all haplotypes from the SC93 population, and of European origin, were clustered into one branch. In another median-joining network built with DNA barcode sequences from China, a shared haplotype existed between the Sichuan and Guizhou populations. Two factors may have contributed to the genetic affinity between some of the gypsy moths from China and those found in Siberia. On the one hand, European gypsy moths may have been introduced into China. On the other hand, Chinese gypsy moths may have been introduced into Europe, and mixed with those already there so went undetected. Even so, we found that the Liaoning moths from 1979 and 1992 showed no genetic affinity with the Siberian samples, in contrast to recent work by Chen et al. (2015). From this, we believed that the spread of gypsy moths between Siberian and Liaoning did not occur earlier than the late 1970s.

Lastly, we found that one haplotype was shared by both the USA96 population and the Asian gypsy moth populations; furthermore, in the phylogenetic tree, these two populations were clustered into one as a single group. Consequently, two factors may have contributed to the genetic affinity between some of the gypsy moths from USA and those found in China. On the one hand, American gypsy moths may have been introduced into China, and mixed with those already there so went undetected. On the other hand, Chinese gypsy moths may have been introduced into USA. This matter could be readily resolved with more molecular data obtained by testing additional samples from these two latter regions. Furthermore, since mtDNA sequences only reflect the movements of females, datasets consisting of both mitochondrial and nuclear sequences are essential to reliably distinguish subspecies of this moth.

Several studies have revealed the genetic structure of gypsy moths, but such analyses have primarily sought to determine the lineage of the gypsy moth in its native range and adjacent areas [24,29,30]. Female gypsy moths from Asia are known for their strong flight ability [13,14,15,16,17]. Moreover, human activities can facilitate moth dispersal to different countries or sites. The female adult will lay eggs on the surface of containers, outdoor furniture, wood, cars, etc., and eggs of gypsy moths in diapause can remain viable for several months without hatching. Human activities provide an opportunity for the transport of egg masses attached to the cargo to a new habitat. For these two reasons, the genetic structure of gypsy moth populations in many parts of its occupied range may have changed quickly. Undoubtedly, the specimens used here were effective in revealing changes in the genetic structure of gypsy moth populations. In order to reveal changes in the genetic structure of additional gypsy moth populations, we encourage other researchers to collect and examine a wider range of molecular data. We only used the *COI* gene sequences, which, when considered alone, are insufficient to reveal further information relating to gypsy moth history, and clearly, in future studies, a wider range of samples and molecular markers are required to reveal additional features of the history and dynamics of this important pest.

## 5. Conclusions

Our analyses of mtDNA *COI* data in gypsy moth specimens from disparate locations revealed previously unknown genetic relationships in populations across space and time. Gypsy moth populations (collected in the same region at different time points) showed relatively high nucleotide polymorphism and genetic diversity. The Beijing gypsy moth population may be more conducive to produce genetic variation at different times based on our results of both nucleotide polymorphism and genetic diversity. We also found that the genetic structure among several Chinese geographical populations in different years was distinct. In addition, the development of international trade and tourism has facilitated genetic flow between gypsy moth populations in parts of China, Japan and Russia. Last but not least, our results suggested that some gypsy moths in China showed the genetic affinity with European gypsy moth (a sub-species of gypsy moth found mainly in Europe).

## Figures and Tables

**Table 1:** *Lymantria dispar* specimens analyzed in this study.

Population Code	Collection Locality	Collection Year	Stage	Type of Preservation	Number of Samples
HN55	Hunan	1955	Adult	Dry	7
HLJ56	Heilongjiang	1956	Adult	Dry	6
NM56	Inner Mongolia	1956	Larva	Formalin	5
DX57	Greater Hinggan	1957	Adult	Dry	10
HB64	Hebei	1964	Adult	Dry	5
HLJ79	Heilongjiang	1979	Adult	Dry	5
LN79	Liaoning	1979	Larva	Formalin	5
BJ79	Beijing	1979	Adult	Dry	8
BJ81	Beijing	1981	Adult	Dry	10
BJ82	Beijing	1982	Adult	Dry	5
BJ87	Beijing	1987	Adult	Dry	6
HLJ92	Heilongjiang	1992	Larva	Formalin	5
SC93	Sichuan	1993	Adult	Dry	10
USA96	USA, Newark, DE	1996	Adult	Dry	8
LN08	Liaoning	2008	Larva	Ethanol	8

**Table 2:** The haplotypes distribution of gypsy moth populations in and around China.

Haplotype Code	Population Code	Country	Prefecture/Province/City/Region	Collection Year	Number of Samples
H1(4)	SHCIQ	China	Yichun, Heilongjiang	2008	1
	LdaRM	Russia	Primorski, far east	2016	1
	LdaTJ	China	Tianjin	2016	1
	LN08	China	Anshan, Liaoning	2008	8
H2(1)	LddNJ	USA	New Jersey	2016	1
H3(1)	LddKG	Greece	Kavála, Macedonia	2016	1
H4(1)	LddLJ	Lithuania	Juodkrante, Kuzsin Nezijos	2016	1
H5(1)	LddKZ	Kazakhstan	Chuy Valley	2016	1
H6(1)	LddRB	Russia	Krasnoyarsk, Siberia	2016	1
H7(2)	LdjJN	Japan	Honshu	2016	1
	LdjID	Japan	Iwate district	2016	1
H8(1)	HN55	China	Dong an, Hunan	1955	6
H9(1)	HN55	China	Dong an, Hunan	1955	1
H10(1)	HLJ56	China	Yichun, dailing, Heilongjiang	1956	4
H11(1)	HLJ56	China	Yichun, dailing, Heilongjiang	1956	2
H12(1)	NM56	China	Caihaer, Inner Mongolia	1956	5
H13(1)	DX57	China	Daxinganling	1957	4
H14(1)	DX57	China	Daxinganling	1957	5
H15(1)	DX57	China	Daxinganling	1957	1
H16(1)	HB64	China	Xinglong, Hebei	1964	3
H17(1)	HB64	China	Xinglong, Hebei	1964	2
H18(1)	HLJ79	China	Yichun, dailing, Heilongjiang	1979	3
H19(1)	HLJ79	China	Yichun, dailing, Heilongjiang	1979	2
H20(1)	BJ79	China	Mentougou, Beijing	1979	8
H21(1)	BJ81	China	Mentougou, Beijing	1981	2
H22(1)	BJ81	China	Mentougou, Beijing	1981	8
H23(1)	BJ82	China	Mentougou, Beijing	1982	2
H24(1)	BJ82	China	Mentougou, Beijing	1982	3
H25(1)	BJ87	China	Xishan, Beijing	1987	6
H26(1)	LN79	China	Anshan, Liaoning	1979	5
H27(1)	HLJ92	China	Heihekou, Heilongjiang	1992	5
H28(1)	SC93	China	Yibin, Sichuan	1993	7
H29(1)	SC93	China	Yibin, Sichuan	1993	1
H30(1)	SC93	China	Yibin, Sichuan	1993	2
H31(1)	USA96	USA	Newark, DE	1996	4
H32(1)	USA96	USA	Newark, DE	1996	4

Note: The number of populations included in each haplotype is given in brackets.

**Table 3:** The haplotypes distribution of gypsy moth populations in China.

Haplotype Code	Population Code	Prefecture/Province/City/Region	Collection Year	Number of Samples
H1(1)	HN55	Dong an, Hunan	1955	6
H2(1)	HN55	Dong an, Hunan	1955	1
H3(1)	NM56	Caihaer, Inner Mongolia	1956	5
H4(1)	DX57	Daxinganling	1957	4
H5(1)	DX57	Daxinganling	1957	5
H6(1)	DX57	Daxinganling	1957	1
H7(1)	HLJ56	Yichun, dailing, Heilongjiang	1956	4
H8(3)	HLJ56	Yichun, dailing, Heilongjiang	1956	2
	LN92	Sitaizi, Liaoning	1992	1
	AX	Arxan, Inner Mongolia	2011	1
H9(3)	AX	Arxan, Inner Mongolia	2011	3
	HLJ79	Yichun, dailing, Heilongjiang	1979	5
	HB12	Longhua, Hebei	2012	1
H10(13)	HLJ92	Qitaihe, Heilongjiang	1992	5
	HLJ12	Heihekou, Heilongjiang	2012	7
	LN92	Anshan, Liaoning	1992	2
	LN08	Anshan, Liaoning	2008	8
	LN11	Sandeli, Liaoning	2011	5
	HB92	Chengde, Hebei	1992	3
	HB12	Longhua, Hebei	2012	8
	BJ87	Xishan, Beijing	1987	6
	BJ92	Labagou, Beijing	1992	2
	AH11	Liu an, Anhui	2011	5
	JN	Jining, Inner Mongolia	2011	5
	JS	Lianyunguang, Jiangsu	2011	10
	AX	Arxan, Inner Mongolia	2011	1
H11(1)	LN79	Anshan, Liaoning	1979	5
H12(1)	LN11	Sandeli, Liaoning	2011	1
	SC93	Yibin, Sichuan	1993	6
	GZ	Xifeng, Guizhou	2011	8
H13(1)	HB64	Xinglong, Hebei	1964	3
H14(1)	HB64	Xinglong, Hebei	1964	2
H15(1)	HB12	Longhua, Hebei	2012	1
H16(1)	BJ79	Mentougou, Beijing	1979	8
H17(1)	BJ81	Mentougou, Beijing	1981	10
	AX	Arxan, Inner Mongolia	2011	1
H18(1)	BJ82	Mentougou, Beijing	1982	2
H19(1)	BJ82	Mentougou, Beijing	1982	3
H20(1)	BJ92	Labagou, Beijing	1992	1
H21(1)	BJ11	Yanzikou, Beijing	2011	9
H22(1)	SC93	Yibin, Sichuan	1993	3
H23(1)	SD92	Laixi, Shandong	1992	2
H24(1)	SD92	Laixi, Shandong	1992	1
H25(1)	AH11	Liu an, Anhui	2011	4
H26(1)	GZ	Xifeng, Guizhou	2011	1
H27(1)	GZ	Xifeng, Guizhou	2011	1
H28(1)	JN	Jining, Inner Mongolia	2011	1
H29(1)	AX	Arxan, Inner Mongolia	2011	1

Note: The number of populations included in each haplotype is given in brackets.

**Table 4:** The haplotypes distribution of gypsy moth populations in the 1990s.

Haplotype Code	Population Code	Prefecture/Province/City/Region	Collection Year	Number of Samples
H1(7)	HLJ92	Qitaihe, Heilongjiang	1992	5
	LN92	Sitaizi, Liaoning	1992	2
	BJ92	Labagou, Beijing	1992	2
	USA96	Newark, DE, USA	1996	4
	HB92	Chengde, Hebei	1992	3
	HS96	Honshu, Japan	1996	6
	PK92	Primorsky Krai, Russia	1992	9
H2(1)	BJ92	Labagou, Beijing	1992	1
H3(1)	LN92	Sitaizi, Liaoning	1992	1
H4(1)	SC93	Yibin, Sichuan	1993	7
H5(1)	SC93	Yibin, Sichuan	1993	3
H6(1)	USA96	Newark, DE, USA	1996	6
H7(1)	SD92	Laixi, Shandong	1992	2
H8(1)	SD92	Laixi, Shandong	1992	1
H9(1)	KL97	Kavala, Greece	1997	3
H10(1)	KL97	Kavala, Greece	1997	5
H11(1)	KL97	Kavala, Greece	1997	1
H12(1)	HS96	Honshu, Japan	1996	1
H13(1)	JK94	Juodkrante, Lithuania	1994	5
H14(1)	KK92	Krasnoyarsk Krai, Russia	1992	5
H15(1)	KK92	Krasnoyarsk Krai, Russia	1992	5
H16(1)	CT94	Connecticut, United States	1994	4
H17(1)	CT94	Connecticut, United States	1994	6

Note: The number of populations included in each haplotype is given in brackets.

## Supplementary Materials

The following are available online at https://www.mdpi.com/2075-4450/10/5/146/s1, Text 1: The *COI* gene sequence obtained in this study, Table S1: All primers used in this study, Table S2: Detailed information of *COI* gene sequences, Table S3: Pairwise *F_ST_* values among Heilongjiang populations, Table S4: Pairwise *F_ST_* values among Liaoning populations, Table S5: Pairwise *F_ST_* values among Hebei populations, Table S6:Pairwise *F_ST_* values among Beijing populations, Table S7: Pairwise *F_ST_* (below the diagonal) and Nm (above the diagonal) values among gypsy moth populations in 1979, Table S8: Pairwise *F_ST_* (below the diagonal) and Nm (above the diagonal) values among gypsy moth populations in 1992, Table S9: Pairwise *F_ST_* (below the diagonal) and Nm (above the diagonal) values among gypsy moth populations in 2011, Table S10: Pairwise *F_ST_* (below the diagonal) and Nm (above the diagonal) values among different populations in the 1950s, Table S11: Pairwise *F_ST_* (below the diagonal) and Nm (above the diagonal) values among different populations in the 1990s.

## References

[1] Ferguson D.C., Dominick R.B., Dominick T., Ferguson D.C., Franclemont J.G., Hodges R.W., Munroe E.G., Classey E.W. (1978). Fascicle 22.2 Noctuoidea, Lymantriidae. The Moths of America North of Mexico.

[2] Pogue M., Schaefer P.W. (2007). Review of selected species of *Lymantria hubner* (1819) (Lepidoptera: Noctuidae: Lymantriinae) from subtropical and temperate regions of Asia, including the descriptions of three new species, some potentially invasive to North America. Int. J. Mech. Control.

[3] Zahiri R., Holloway J.D., Kitching I.J., Lafontaine J.D., Mutanen M., Wahlberg N. (2011). Molecular phylogenetics of Erebidae (Lepidoptera, Noctuoidea). Syst. Entomol..

[4] Chen N.Z. (2013). Bioecology of gypsy moth. Gypsy Moth Quarantine and Control.

[5] Montgomery M.E., Wallner W.E., Berryman A.A. (1988). The gypsy moth a westward migrant. Dynamics of Forest Insect Populations, Patterns, Causes, Implications.

[6] Pimentel D., Zuniga R., Morrison D. (2005). Update of the environmental and economic costs associated with alien-invasive species in the United States. Ecol. Econ..

[7] Xiao G.R., Xu W.R. (1992). Lymantridae. Chinese Forest Insect.

[8] Forbush E.H., Fernald C.H. (1896). The Gypsy Moth.

[9] Giese R.L., Schneider M.L. (1979). Cartographic comparisons of Eurasian gypsy moth distribution (*Lymantria dispar*.; Lepidoptera: Lymantriidae). Entomol. News.

[10] Reineke A., Karlovsky P., Zebitz C.P.W. (1999). Amplified fragment length polymorphism analysis of different geographic populations of the gypsy moth, *Lymantria dispar* (Lepidoptera: Lymantriinae). Bull. Entomol. Res..

[11] Zhou X., Chen N., Yang D. (2009). Research advances in the population genetic relationships of *Lymantria dispar* Linnaeus based on molecular genetic markers. Plant Prot..

[12] Djoumad A., Nisole A., Zahiri R., Freschi L., Picq S., Gundersenrindal D.E. (2017). Comparative analysis of mitochondrial genomes of geographic variants of the gypsy moth, *Lymantria dispar*, reveals a previously undescribed genotypic entity. Sci. Rep..

[13] Keena M.A., Grinberg P.S., Wallner W.E. (2007). Inheritance of female flight in *Lymantria dispar* (Lepidoptera: Lymantriidae). Environ. Entomol..

[14] Keena M.A., Côté M., Grinberg P.S., Wallner W.E. (2008). World distribution of female flight and genetic variation in *Lymantria dispar* (Lepidoptera: Lymantriidae). Environ. Entomol..

[15] Yang F. (2013). The Study on Flight Ability among Different Geographic Populations of Asian Gypsy Moth, *Lymantria dispar* in China. Ph.D. Thesis.

[16] Shi J., Chen F., Keena M.A. (2015). Differences in wing morphometrics of *Lymantria dispar* (Lepidoptera: Erebidae) between populations that vary in female flight capability. Ann. Entomol. Soc. Am..

[17] Chen F., Shi J., Keena M. (2016). Evaluation of the effects of light intensity and time interval after the start of scotophase on the female flight propensity of Asian gypsy moth (Lepidoptera: Erebidae). Environ. Entomol..

[18] Wallner W.E., Humble L.M., Levin R.E., Baranchikov Y.N., Cardé R.T. (1995). Response of adult Lymantriid moths to illumination devices in the Russian Far East. J. Econ. Entomol..

[19] Iwaizumi R., Arakawa K. (2010). Report on female flight activity of the Asian gypsy moth, *Lymantria dispar* (Lepidoptera: Lymantriidae) and flight suppression with a yellow light source in Japan. Res. Bull. Plant Prot. Serv. Jpn..

[20] Dewaard J.R., Mitchell A., Keena M.A., Gopurenko D., Boykin L.M., Armstrong K.F. (2010). Towards a global barcode library for *Lymantria* (Lepidoptera: Lymantriinae) tussock moths of biosecurity concern. PLoS ONE.

[21] Bogdanowicz S.M., Mastro V.C., Prasher D.C., Harrison R.G. (1997). Microsatellite DNA variation among Asian and North American gypsy moths (Lepidoptera: Lymantridae). Ann. Entomol. Soc. Am..

[22] Bogdanowicz S.M., Schaefer P.W., Harrison R.G. (2000). Mitochondrial DNA variation among worldwide populations of gypsy moths, *Lymantria dispar*. Mol. Phylogenet. Evol..

[23] Chen F., Shi J., Luo Y.Q. (2013). Genetic characterization of the gypsy moth from China (Lepidoptera, Lymantriidae) using inter simple sequence repeats markers. PLoS ONE.

[24] Chen F., Luo Y., Keena M.A., Wu Y., Wu P., Shi J. (2015). DNA barcoding of gypsy moths from China (Lepidoptera: Erebidae) reveals new haplotypes and divergence patterns within gypsy moth subspecies. J. Econ. Entomol..

[25] Kang T.H., Han S.H., Park S.J. (2015). Development of seven microsatellite markers using next generation sequencing for the conservation on the Korean population of *Dorcus hopei* (*E. Saunders*, 1854) (Coleoptera, Lucanidae). Int. J. Mol. Sci..

[26] Kang T.H., Han S.H., Park S.J. (2016). Development of 12 microsatellite markers in *Dorcus titanus castanicolor* (Motschulsky, 1861) (Lucanidae, Coleoptera) from Korea using next-generation sequencing. Int. J. Mol. Sci..

[27] Qian L. (2011). AFLP analysis of different geographic populations of the gypsy moth, *Lymantria dispar* (Lepidoptera: Lymantriidae). Sci. Silvae Sin..

[28] Zhang H., Chen N.Z., Li Z.X. (2011). Analysis of RAPD markers and development of SCAR markers of six geographic populations of the gypsy moth, *Lymantria dispar* L. (Lepidoptera: Lymantriidae), from China. Acta Entomol. Sin..

[29] Wu Y., Molongoski J.J., Winograd D.F., Bogdanowicz S.M., Louyakis A.S., Lance D.R. (2015). Genetic structure, admixture and invasion success in a Holarctic defoliator, the gypsy moth (*Lymantria dispar*, Lepidoptera: Erebidae). Mol. Ecol..

[30] Kang T.H., Sang H.H., Lee H.S. (2017). Genetic structure and demographic history of *Lymantria dispar* (Linnaeus, 1758) (Lepidoptera: Erebidae) in its area of origin and adjacent areas. Ecol. Evol..

[31] Lee K.S., Kang T.H., Jeong J.W., Ryu D.P., Lee H.S. (2015). Taxonomic review of the genus *Lymantria* (Lepidoptera: Erebidae: Lymantriinae) in Korea. Entomol. Res..

[32] Wandeler P., Hoeck P.E.A., Keller L.F. (2007). Back to the future: Museum specimens in population genetics. Trends Ecol. Evol..

[33] Ewers R.M., Didham R.K. (2006). Confounding factors in the detection of species responses to habitat fragmentation. Biol. Rev..

[34] Lister A.M. (2011). Natural history collections as sources of long-term data sets. Trends Ecol. Evol..

[35] Holmes M.W., Hammond T.T., Wogan G.O., Walsh R.E., LaBarbera K., Wommack E.A. (2016). Natural history collections as windows on evolutionary processes. Mol. Ecol..

[36] Krehenwinkel H., Pekar S. (2015). An analysis of factors affecting genotyping success from museum specimens reveals an increase of genetic and morphological variation during a historical range expansion of a European spider. PLoS ONE.

[37] Mikheyev A.S., Tin M.M., Arora J., Seeley T.D. (2015). Museum samples reveal rapid evolution by wild honey bees exposed to a novel parasite. Nat. Commun..

[38] Hajibabaei M., Smith M.A., Janzen D.H., Rodriguez J.J., Whitfield J.B., Hebert P.D.N. (2006). A minimalist barcode can identify a specimen whose DNA is degraded. Mol. Ecol. Notes.

[39] Rowley D.L., Coddington J.A., Gates M.W., Norrbom R.A., Ochoa R., Vandenberg N.J. (2007). Vouchering DNA-barcoded specimens: Test of a nondestructive extraction protocol for terrestrial arthropods. Mol. Ecol. Notes.

[40] Meusnier I., Singer G.A., Landry J.F., Hickey D.A., Hebert P.D., Hajibabaei M. (2008). A universal DNA mini-barcode for biodiversity analysis. BMC Genom..

[41] Zimmermann J., Hajibabaei M., Blackburn D.C., Hanken J., Cantin E., Posfai J. (2008). DNA damage in preserved specimens and tissue samples: A molecular assessment. Front. Zool..

[42] Hausmann A., Herbert P.D., Mitchell A., Rougerie R., Sommerer M., Edwards T. (2009). Revision of the Australian *Oenochroma vinaria* Guenée, 1858 species-complex (Lepidoptera: Geometridae, Oenochrominae): DNA barcoding reveals cryptic diversity and assesses status of type specimen without dissection. Zootaxa.

[43] Espeland M., Irestedt M., Johanson K.A., Akerlund M., Bergh J.E., Källersjö M. (2010). Dichlorvos exposure impedes extraction and amplification of DNA from insects in museum collections. Front. Zool..

[44] Nagy Z.T., Breman F.C., Dall’Asta U. (2010). DNA barcoding of museum specimens of Lymantriidae preserved in the Royal Museum for Central Africa. Entomol. Roman..

[45] Welch A.J., Wiley A.E., James H.F., Ostrom P.H., Stafford S.T., Fleischer R.C. (2012). Ancient DNA reveals genetic stability despite demographic decline: 3000 years of population history in the endemic Hawaiian petrel. Mol. Biol. Evol..

[46] Miller J.A., Beentjes K.K., Helsdingen P., Ijland S. (2013). Which specimens from a museum collection will yield DNA barcodes? A time series study of spiders in alcohol. Zookeys.

[47] Hernández-Triana L.M., Prosser S., Rodríguez-Perez M.A., Chaverri L.G., Hebert P.D., Gregory T.R. (2014). Recovery of DNA barcodes from blackfly museum specimens (Diptera: Simuliidae) using primer sets that target a variety of sequence lengths. Mol. Ecol. Resour..

[48] Malmström H., Linderholm A., Skoglund P., Storå J., Sjödin P., Gilbert M.T. (2015). Ancient mitochondrial DNA from the Northern fringe of the neolithic farming expansion in Europe sheds light on the dispersion process. Philos. Trans. R. Soc. Biol. Sci..

[49] Price B.W., Henry C.S., Hall A.C., Mochizuki A., Duelli P., Brooks S.J. (2015). Singing from the grave: DNA from a 180-year old type specimen confirms the identity of *Chrysoperla carnea* (Stephens). PLoS ONE.

[50] White L.C., Mitchell K.J., Austin J.J. (2018). Ancient mitochondrial genomes reveal the demographic history and phylogeography of the extinct, enigmatic Thylacine (*Thylacinus cynocephalus*). J. Biogeogr..

[51] Alzohairy A.M. (2011). Bioedit: An important software for molecular biology. GERF Bull. Biosci..

[52] Whitlock M.C., Mccauley D.E. (1999). Indirect measures of gene flow and migration: *F*_ST_ not equal to 1/(4nm + 1). Heredity.

[53] Excoffier L., Laval G., Schneider S. (2005). Arlequin ver. 3.0: An integrated software package for population genetics data analysis. Evol. Bioinform. Online.

[54] Bandelt H.J., Forster P., Röhl A. (1999). Median-joining networks for inferring intraspecific phylogenies. Mol. Biol. Evol..

[55] Librado P., Rozas J. (2009). Dnasp v5: A software for comprehensive analysis of DNA polymorphism data. Bioinformatics.

[56] Kumar S., Stecher G., Tamura K. (2016). Mega 7: Molecular evolutionary genetics analysis version 7.0 for bigger datasets. Mol. Biol. Evol..

[57] Feng G.E. (2011). Challenges facing entomologists in a changing global climate. Chin. J. Appl. Entomol..

