# Supplementary material for: Mitochondrial Gene Sequence (COI) Reveals the Genetic Structure and Demographic History of Lymantria dispar (Lepidoptera: Erebidae: Lymantriinae) in and around China
Source: Insects. 2019 May 22;10(5):146. doi: 10.3390/insects10050146 (PMC6572239; doi:10.3390/insects10050146)
Supplement: Supplementary file 1 [file insects-10-00146-s001.zip › Supplementary Materials/Table S1-S11.doc]

| **Table S1** All primers used in this study | | | | | |
| --- | --- | --- | --- | --- | --- |
| **Primers Name** | **Direction** | **Nucleotide sequence 5'-3'** | **Position (Min-Max)** | **Length (bp)** | **Anneal temperatures** |
| Lco 1 | F | GCATGAGCTGTAACAATAG | 1412-1628 | 216 | 49.6 |
|  | R | CAATTTATCGCTTAAACTCAGCC |  |  |  |
| Lco 2 | F | CGTCGAGGTATACCTGCTAATCC | 281-553 | 272 | 55.4 |
|  | R | CACTGTAGGAGGATTAACTGGAG |  |  |  |
| Lco 3 | F | AATCAATTACCAAATCCTCC | 1352-1625 | 273 | 48 |
|  | R | TTTACCGCTTAAACTCAGC |  |  |  |
| Lco 4 | F | CCAATTACCAAATCCTCC | 1354-1628 | 274 | 48 |
|  | R | CAATTTATCGCTTAAACTCAGCC |  |  |  |
| Lco 5 | F | CCTAATCTTCAGAGAATAGAAGGTGAGT | 569-852 | 283 | 53.4 |
|  | R | CCAGGATTTGGAATAATTTCCC |  |  |  |
| Lco 6 | F | GGATAATCCTTTATGAATGGGG | 1-285 | 285 | 51 |
|  | R | CGACGATACTCTGATTACCCAG |  |  |  |
| Lco 7 | F | GAACGTAGTGGAAATGAGCTAC | 455-747 | 292 | 52.3 |
|  | R | GGATTTATTGTTTGGGCTCACC |  |  |  |
| Lco 8 | F | GTAGCAGAGGTAAAGTAAGCTCG | 627-926 | 299 | 54.3 |
|  | R | GGAGGAGGGGATCCAATCCT |  |  |  |
| Lco 9 | F | CCTGAAAATAGTGGATATCAGTG | 392-695 | 303 | 54.3 |
|  | R | CACGAGCTTACTTTACCTCTGC |  |  |  |
| Lco 10 | F | GCAGGGTCAAAAAAGG | 929-1237 | 308 | 48 |
|  | R | TGGAGCAGGAACAGGA |  |  |  |
| Lco 11 | F | GGGGGAAAAGAGCAATTCGTTG | 140-460 | 320 | 52.3 |
|  | R | CGTTCTTTCTATAGGAGCAG |  |  |  |
| Lco 12 | F | GGATCCCCTCCTCCTGCAGGGTC | 914-1236 | 322 | 56.8 |
|  | R | GGAGCAGGAACAGGATGAACTG |  |  |  |
| Lco 13 | F | GCAGAGGTAAAGTAAGCTCGTG | 674-1004 | 330 | 53.4 |
|  | R | CTTTACCTGTTTTAGCAGGTGC |  |  |  |
| Lco 14 | F | GAGGTGATGCTCTATTTTGAAG | 138-480 | 342 | 51 |
|  | R | CGACGATACTCTGATTACCCAG |  |  |  |
| Lco 15 | F | CCAGCTAAGTGAAGAGAAA | 1136-1486 | 350 | 48.5 |
|  | R | TCGAGCTGAATTAGGGAAT |  |  |  |
| Lco 16 | F | GCACCTGCTAAAACAGGTAAAG | 983-1335 | 352 | 51 |
|  | R | GGAGCCCCTGATATAGCTTTCCCCCG |  |  |  |
| Lco 17 | F | TCATCCTGTTCCTGCTCCATTTTC | 1219-1572 | 353 | 52.3 |
|  | R | TCAACAAATCATAAGGATATTGGAACC |  |  |  |
| Lco 18 | F | GGGAAATTATTCAAATCCTGG | 831-1188 | 357 | 49.6 |
|  | R | GCTCATGGAGGTAGATCTGTTG |  |  |  |
| Lco 19 | F | GATGAAATACCAGCTAAGTGAAGAG | 1127-1485 | 358 | 51 |
|  | R | CGAGCTGAATTAGGGAATCCTGGATC |  |  |  |
| Lco 20 | F | CCTAATCTTCAGAGAATAGAAGGTGAGT | 569-936 | 367 | 53.4 |
|  | R | GACCCTGCAGGAGGAGGGGATCCAATCC |  |  |  |
| Lco 21 | F | TCAGAGAATAGAAGGTGAGT | 577-948 | 371 | 48.5 |
|  | R | ACATCCTTTTTTGACCCTG |  |  |  |
| Lco 22 | F | CCAGCTAAGTGAAGAGAAA | 1136-1522 | 386 | 48 |
|  | R | ATCAGGAATAGTGGGAAC |  |  |  |
| Lco 23 | F | TGGGAAATTATTCCAAATCCTGG | 830-1424 | 412 | 48.5 |
|  | R | GAAAATGGAGCAGGAACAGGATGA |  |  |  |
| Lco 24 | F | TTTTTCCTCTTTCTTGGG | 804-1224 | 420 | 48.5 |
|  | R | GGATGAACTGTTTACCCTC |  |  |  |
| Lco 25 | F | GGGTAATCAGAGTATCGTCG | 266-743 | 477 | 48.5 |
|  | R | TTATTGTTTGGGCTCACCAC |  |  |  |
| Lco 26 | F | GAGCCCAAACAATAAATC | 729-1237 | 508 | 48.5 |
|  | R | TGGAGCAGGAACAGGA |  |  |  |
| Lco 27 | F | TGGTGAGCCCAAACAATAAA | 725-1241 | 516 | 48.5 |
|  | R | AAAATGGAGCAGGAACAGGA |  |  |  |
| Lco 28 | F | GCAGGGTCAAAAAAGGAT | 929-1486 | 557 | 48.5 |
|  | R | TCGAGCTGAATTAGGGAA |  |  |  |
| Lco 29 | F | TCAGAGAATAGAAGGTGAGT | 577-1247 | 670 | 48.5 |
|  | R | TTGTAGAAAATGGAGCAG |  |  |  |
| Lco 30 | F | GGGTAATCAGAGTATCGTCG | 266-1243 | 977 | 48.5 |
|  | R | AGAAAATGGAGCAGGAACAG |  |  |  |
| Lco 31 | F | TTTAGGGGGAAAAGAGGA | 137-1239 | 1102 | 48.5 |
|  | R | AATGGAGCAGGAACAGGA |  |  |  |

**Table S2** The detailed information of COI gene sequences

| **Populations code** | **Country** | **Prefecture/Province/City/Region** | **Collected time** | **Reference** | **COI-5P BOLD ID/Genbank ID** |
| --- | --- | --- | --- | --- | --- |
| KL97 | Greece | Kavala | Feb, 1997 | Chen et al. 2016 | GMCF092-14~GMCF100-14 |
| HS96 | Japan | Honshu | Mar,1996 | Chen et al. 2016 | GMCF075-14,GMCF085-14~GMCF091-14 |
| JK94 | Lithuania | Juodkrante | Aug,1994 | Chen et al. 2016 | GMCF101-14~GMCF105-14 |
| KK92 | Russia | Krasnoyarsk Krai | Dec,1992 | Chen et al. 2016 | GMCF116-14~GMCF125-14 |
| PK92 | Russia | Primorsky Krai | Aug,1992 | Chen et al. 2016 | GMCF126-14~GMCF134-14 |
| CT94 | United States | Conneticut | Mar,1994 | Chen et al. 2016 | GMCF106-14~GMCF115-14 |
| BJ92 | China | Beijing | 1992 | de Waard et al 2010 | HM775690~HM775692 |
| HB92 | China | Hebei | 1992 | de Waard et al 2010 | HM775687~HM775689 |
| LN92 | China | Liaoning | 1992 | de Waard et al 2010 | HM775686,HM775607~HM775608 |
| SD92 | China | Shandong | 1992 | de Waard et al 2010 | HM775605, HM775684, HM775685 |
| AH | China | Anhui | Aug,2011 | Chen et al. 2016 | GMCF001-14~GMCF009-14 |
| BJ11 | China | Beijing | Aug,2011 | Chen et al. 2016 | GMCF010-14~GMCF018-14 |
| GZ | China | Guizhou | Nov,2011 | Chen et al. 2016 | GMCF019-14~GMCF028-14 |
| HB12 | China | Hebei | Mar,2012 | Chen et al. 2016 | GMCF029-14~GMCF038-14 |
| JN | China | Jining | Aug,2011 | Chen et al. 2016 | GMCF039-14~GMCF044-14 |
| LN11 | China | Liaoning | Sept,2011 | Chen et al. 2016 | GMCF045-14~GMCF050-14 |
| HLJ12 | China | Heilongjiang | Aug,2012 | Chen et al. 2016 | GMCF051-14~GMCF057-14 |
| JS | China | Jiangsu | July,2011 | Chen et al. 2016 | GMCF058-14~GMCF067-14 |
| AX | China | Arxan | June, 2011 | Chen et al. 2016 | GMCF068-14~GMCF074-14 |
| SHCIQ | China | Heilongjiang | 2008 | Zhu et al, 2010 | FJ617240 |
| LddNJ | USA | New Jersey |  | Djoumad, et al,2017 | KY798442 |
| LddKG | Greece | Kavála, Macedonia |  | Djoumad, et al,2017 | KY923062 |
| LddLJ | Lithuania | Juodkrante, Kuzsin Nezijos |  | Djoumad, et al,2017 | KY923063 |
| LddKZ | Kazakhstan | Chuy Valley， |  | Djoumad, et al,2017 | KY923065 |
| LddRB | Russia | Krasnoyarsk, Siberia |  | Djoumad, et al,2017 | KY923064 |
| LdaTJ | China | Tianjin |  | Djoumad, et al,2017 | KY923067 |
| LdaRM | Russia | Primorski, far east |  | Djoumad, et al,2017 | KY923059 |
| LdjJN | Japan | Honshu |  | Djoumad, et al,2017 | KY923061 |
| LdjID | Japan | Iwate district |  | Djoumad, et al,2017 | KY923060 |
| LuJH | Japan | Hokkaido |  | Djoumad, et al,2017 | KY923066 |

| **References** |  |
| --- | --- |
| Chen *et al*., 2015 | Chen, F., Luo, Y., Keena, M.A., Wu, Y., Wu, P., Shi, J. DNA barcoding of gypsy moths from China (Lepidoptera: Erebidae) reveals new haplotypes and divergence patterns within gypsy moth subspecies. *Journal of Economic Entomology* **2015**, *109*, 366-374. |
| DeWaard *et al*., 2010 | Dewaard, J.R.; Mitchell, A.; Keena, M.A, Gopurenko, D.; Boykin, LM.; Armstrong, K.F. Towards a global barcode library for *Lymantria* (Lepidoptera: Lymantriinae) tussock moths of biosecurity concern. *PloS One* **2010**, *5*, e14280. |
| Djoumad *et al*., 2017 | Djoumad, A.; Nisole, A.; Zahiri, R.; Freschi, L.; Picq, S.; Gundersenrindal, D.E. Comparative analysis of mitochondrial genomes of geographic variants of the gypsy moth, *Lymantria dispar*, reveals a previously undescribed genotypic entity. *Scientific Reports* **2017**, *7*, 14245. |
| Zhu *et al*., 2010 | Zhu, Y.J.; Zhou, G.L.; Fang, R.; Ye. J.; Yi., J.P. The complete sequence determination and analysis of *Lymantria dispar* (Lepidoptera: Lymantriidae) mitochondrial genome. *Plant Quarantine* **2010**, *24,* 6-11. |

**Table S3** Pairwise *F*_ST_ values among Heilongjiang populations

|  | HLJ56 | HLJ79 | HLJ92 |
| --- | --- | --- | --- |
| HLJ56 |  |  |  |
| HLJ79 | 0.9196^＋^ |  |  |
| HLJ92 | 0.8897^＋^ | 1.0000^＋^ |  |
| HLJ12 | 0.9085^＋^ | 1.0000^＋^ | 0.0000^－^ |
| Significant differences: ＋,Non-significant difference:－ | | | |

Table S4 Pairwise *F*_ST_ values among Liaoning populations

|  | LN79 | LN92 | LN08 |
| --- | --- | --- | --- |
| LN79 |  |  |  |
| LN92 | 0.6508^＋^ |  |  |
| LN08 | 1.0000^＋^ | 0.7277^＋^ |  |
| LN11 | 0.5114^＋^ | 0.6047^－^ | 0.0514^－^ |
| Significant differences: ＋,Non-significant difference:－ | | | |

Table S5 Pairwise *F*_ST_ values among Hebei populations

|  | HB64 | HB92 | HB12 |
| --- | --- | --- | --- |
| HB64 |  |  |  |
| HB92 | 0.4915－ |  |  |
| HB12 | 0.5847＋ | 0.1842－ | 0.0000－ |
| Significant differences: ＋, Non-significant difference:－ | | | |

Table S6 Pairwise *F*_ST_ values among Beijing populations

|  | BJ79 | BJ81 | BJ82 | BJ87 | BJ92 |
| --- | --- | --- | --- | --- | --- |
| BJ79 |  |  |  |  |  |
| BJ81 | 0.9124^＋^ |  |  |  |  |
| BJ82 | 0.9498^＋^ | 0.8148^＋^ |  |  |  |
| BJ87 | 0.8510^＋^ | 0.6659^＋^ | 0.7450^＋^ |  |  |
| BJ92 | 0.9334^＋^ | 0.6735^＋^ | 0.7719^＋^ | 0.1692^－^ |  |
| BJ11 | 1.0000^＋^ | 0.8830^＋^ | 0.9399^＋^ | 0.7738^＋^ | 0.8902^＋^ |
| Significant differences: ＋, Non-significant difference:－ | | | | | |

**Table S7 Pairwise *F*_ST_( below the diagonal) and Nm (above the diaognal) values among Heilongjiang populations in 1979**

|  | HLJ79 | LN79 | BJ79 |
| --- | --- | --- | --- |
| HLJ79 |  | 0.0500 | 0.0716 |
| LN79 | 0.9091^＋^ |  | 0.0000 |
| BJ79 | 0.8748^＋^ | 1.0000^＋^ |  |
| Significant differences: ＋,Non-significant difference:－ | | | |

Table S8 Pairwise *F*_ST_ ( below the diagonal) and Nm (above the diaognal) values among Heilongjiang populations in 1992

|  | HLJ92 | LN92 | BJ92 |
| --- | --- | --- | --- |
| HLJ92 |  | 0.2997 | 2.1429 |
| LN92 | 0.6252^＋^ |  | 0.5455 |
| BJ92 | 0.1892^－^ | 0.4783－ |  |
| Significant differences: ＋,Non-significant difference:－ | | | |

Table S9 Pairwise *F*_ST_ (below the diagonal) and Nm (above the diaognal) values among Heilongjiang populations in 2011

|  | HLJ12 | LN12 | BJ11 |
| --- | --- | --- | --- |
| HLJ12 |  | 17.5000 | 0.0000 |
| LN12 | 0.0278^－^ |  | 0.3053 |
| BJ11 | 1.0000^＋^ | 0.6209^＋^ |  |
| Significant differences: ＋,Non-significant difference:－ | | | |

Table S10 Pairwise *F*_ST_ ( below the diagonal) and Nm (above the diaognal) values among different populations in 1950s

|  | HN55 | HLJ56 | NM56 | DX57 |
| --- | --- | --- | --- | --- |
| HN55 |  | 0.0513 | 0.0344 | 0.0652 |
| HLJ56 | 0.9069^＋^ |  | 0.0620 | 0.0507 |
| NM56 | 0.9356^＋^ | 0.8897^＋^ |  | 0.0417 |
| DX57 | 0.8847^＋^ | 0.9079^＋^ | 0.9231^＋^ |  |
| Significant differences: ＋,Non-significant difference:－ | | | | |

**Table S11** Pairwise *F*_ST_ (below the diagonal) and Nm (above the diagonal) values among different populations in 1990s

|  | **HLJ92** | **LN92** | **BJ92** | **SC93** | **USA96** | **HB92** | **SD92** | **KL97** | **HS96** | **JK94** | **KK92** | **PK92** | **CT94** |
| --- | --- | --- | --- | --- | --- | --- | --- | --- | --- | --- | --- | --- | --- |
| HLJ92 |  | 0.2997 | 2.1429 | 0.0832 | 0.9524 | inf | 1.6011 | 0.1276 | 0.0000 | inf | 0.0459 | 0.0000 | 0.0302 |
| LN92 | 0.6252^＋^ |  | 0.5455 | 0.1428 | 0.2284 | 0.5227 | 63.0000 | 0.1815 | 0.2262 | 0.1937 | 0.1340 | 0.1665 | 0.1150 |
| BJ92 | 0.1892^－^ | 0.4783^－^ |  | 0.1135 | 1.3884 | inf | 10.0000 | 0.1696 | 4.6667 | 0.0184 | 0.0680 | 0.8182 | 0.0453 |
| SC93 | 0.8573^＋^ | 0.7778^＋^ | 0.8151^＋^ |  | 0.1174 | 0.1001 | 0.2662 | 0.2090 | 0.0860 | 0.0721 | 0.1351 | 0.0629 | 0.0786 |
| USA96 | 0.3443^－^ | 0.6864^＋^ | 0.2648^－^ | 0.8098^＋^ |  | 1.5000 | 0.8837 | 0.1450 | 0.8889 | 0.0570 | 0.0872 | 0.6102 | 0.0718 |
| HB92 | 0.0000^－^ | 0.4889^－^ | 0.0000^－^ | 0.8332^＋^ | 0.2500^－^ |  | 9.5000 | 0.1575 | 0.0000 | 0.0000 | 0.0547 | inf | 0.0360 |
| SD92 | 0.2380^＋^ | 0.0079^－^ | 0.0476^－^ | 0.6526^＋^ | 0.3614^＋^ | 0.0500^－^ |  | 0.3303 | 1.0184 | 0.3022 | 0.2392 | 0.6732 | 0.1784 |
| KL97 | 0.7967^＋^ | 0.7337^＋^ | 0.7467^＋^ | 0.7052^＋^ | 0.7752^＋^ | 0.7604^＋^ | 0.6022^＋^ |  | 0.1214 | 0.4919 | 0.6798 | 0.0939 | 0.3930 |
| HS96 | 0.0553^－^ | 0.6885^＋^ | 0.0968^－^ | 0.8532^＋^ | 0.3600^－^ | 0.1667^－^ | 0.3293^＋^ | 0.8047^＋^ |  | 0.0143 | 0.0531 | 12.6000 | 0.0354 |
| JK94 | 1.0000^＋^ | 0.7208^＋^ | 0.9646^＋^ | 0.8740^＋^ | 0.8977^＋^ | 1.0000^＋^ | 0.6233^＋^ | 0.5041^＋^ | 0.9722^＋^ |  | 0.0877 | 0.0000 | 0.0877 |
| KK92 | 0.9160^＋^ | 0.7887^＋^ | 0.8804^＋^ | 0.7873^＋^ | 0.8515^＋^ | 0.9013^＋^ | 0.6764^＋^ | 0.4238^＋^ | 0.9040^＋^ | 0.8508^＋^ |  | 0.0349 | 0.1156 |
| PK92 | 0.0000^－^ | 0.7502^＋^ | 0.3793^－^ | 0.8883^＋^ | 0.4504^＋^ | 0.0000^－^ | 0.4262^＋^ | 0.8418^＋^ | 0.0382^－^ | 1.0000^＋^ | 0.9348^＋^ |  | 0.0230 |
| CT94 | 0.9430^＋^ | 0.8130^＋^ | 0.9169^＋^ | 0.8642^＋^ | 0.8744^＋^ | 0.9329^＋^ | 0.7370^＋^ | 0.5599^＋^ | 0.9338^＋^ | 0.8508^＋^ | 0.8123^＋^ | 0.9560^＋^ |  |

Significant differences: ＋, Non-significant difference:－, inf: Nm values cannot be calculated because the FST values are zero.
